# Serosurvey of tuberculosis in wild boars in Xinjiang, Northwest China: a pilot study

**DOI:** 10.1186/s13620-026-00334-6

**Published:** 2026-02-04

**Authors:** Jian-Yong Wu, Xiao-Xiao Meng, Hongduzi Bolati, Xue-Yun Yang

**Affiliations:** 1 Key Laboratory of Environmental Exposure Omics, School of Public Health, Institute of Medical Sciences of Xinjiang Medical University, Xinjiang Medical University, Urumqi, China; 2 Xinjiang Key Laboratory of Animal Infectious Diseases, Institute of Veterinary Medicine, Xinjiang Academy of Animal Sciences, Urumqi, 830013 China

## Abstract

**Background:**

Tuberculosis (TB), caused by *Mycobacterium tuberculosis* complex (MTBC), poses a significant risk for zoonotic transmission, especially with the increasing wild boar population in China. Following the annual wildlife disease surveillance, we conducted a serosurvey to determine the prevalence of TB in wild boars. We collected 512 serum samples from 25 counties of Xinjiang, Northwest China, and used ELISA to detect antibodies against bPPD. TB seropositivity was defined as a mean sample OD450nm ≥ 2×the mean negative control OD450nm, and employed cluster analysis with purely spatial scan statistics using the Bernoulli model.

**Results:**

The seropositivity was 10.0% (51/512), with higher rates in farmed wild boars (12.0%, 47/391) than feral wild boars (3.3%, 4/121), indicating a greater risk in farmed wild boar populations. Spatial analysis revealed two clusters near the Sino-Kazakh border, underscoring the need for targeted surveillance and control measures to prevent zoonotic transmission.

**Conclusion:**

Xinjiang is a endemic area for MTBC exposure, with farmed wild boars posing higher risk. Findings highlight the need for targeted surveillance, control measures, and international collaboration to prevent zoonotic transmission.

## Introduction

 Tuberculosis (TB), a chronic infectious disease caused by the *Mycobacterium tuberculosis* complex (MTBC) (including *Mycobacterium tuberculosis*, *Mycobacterium bovis*, and related species), remains a major global health concern affecting both human and animal populations [1, 2]. While human TB has been extensively studied, zoonotic TB, particularly its epidemiology in wildlife reservoirs, has gained increasing attention in recent years [3–5]. Among animal species susceptible to MTBC infection, wild boars (*Sus scrofa*) have emerged as significant hosts, playing a potential role in the maintenance and transmission of TB at the wildlife-livestock-human interface [6].

In China, the wild boar population has experienced substantial growth due to ecological restoration initiatives, changes in land use patterns, and the implementation of wildlife protection policies [7–9]. This population expansion, coupled with the species’ wide geographical distribution and behavioral adaptability, has led to an increased frequency of interactions among wild boars, domestic animals, and human populations [10, 11]. Such ecological changes raise substantial concerns about the potential for interspecies transmission of zoonotic pathogens, particularly MTBC, which represents a significant threat to both public health and livestock industries.

Current epidemiological data indicate the presence of MTBC in wild boar populations across multiple countries, with reported prevalence rates varying from 2% to 50% depending on geographical location and study methodology [5, 12]. These findings underscore the importance of wild boars as potential maintenance hosts in the epidemiology of animal TB. In China, while TB in domestic livestock (including cattle, sheep, and pigs) has been well-documented through national surveillance programs [13, 14], the epidemiological status of TB in wild boar populations remains poorly understood. This knowledge gap is particularly concerning given the species’ ecological plasticity and its increasing proximity to human settlements and agricultural areas.

The objective of this study is to conduct a serosurvey of TB in wild boars across various regions in Xinjiang, China. We aim to determine the seroprevalence of TB in these populations and identify any potential risk factors associated with infection. This information is essential for developing interventions to mitigate the spread of TB in both wildlife and domestic animals, thereby reducing the zoonotic risk and contributing to the overall goal of TB control and elimination.

## Materials and methods

**Fig. 1:**
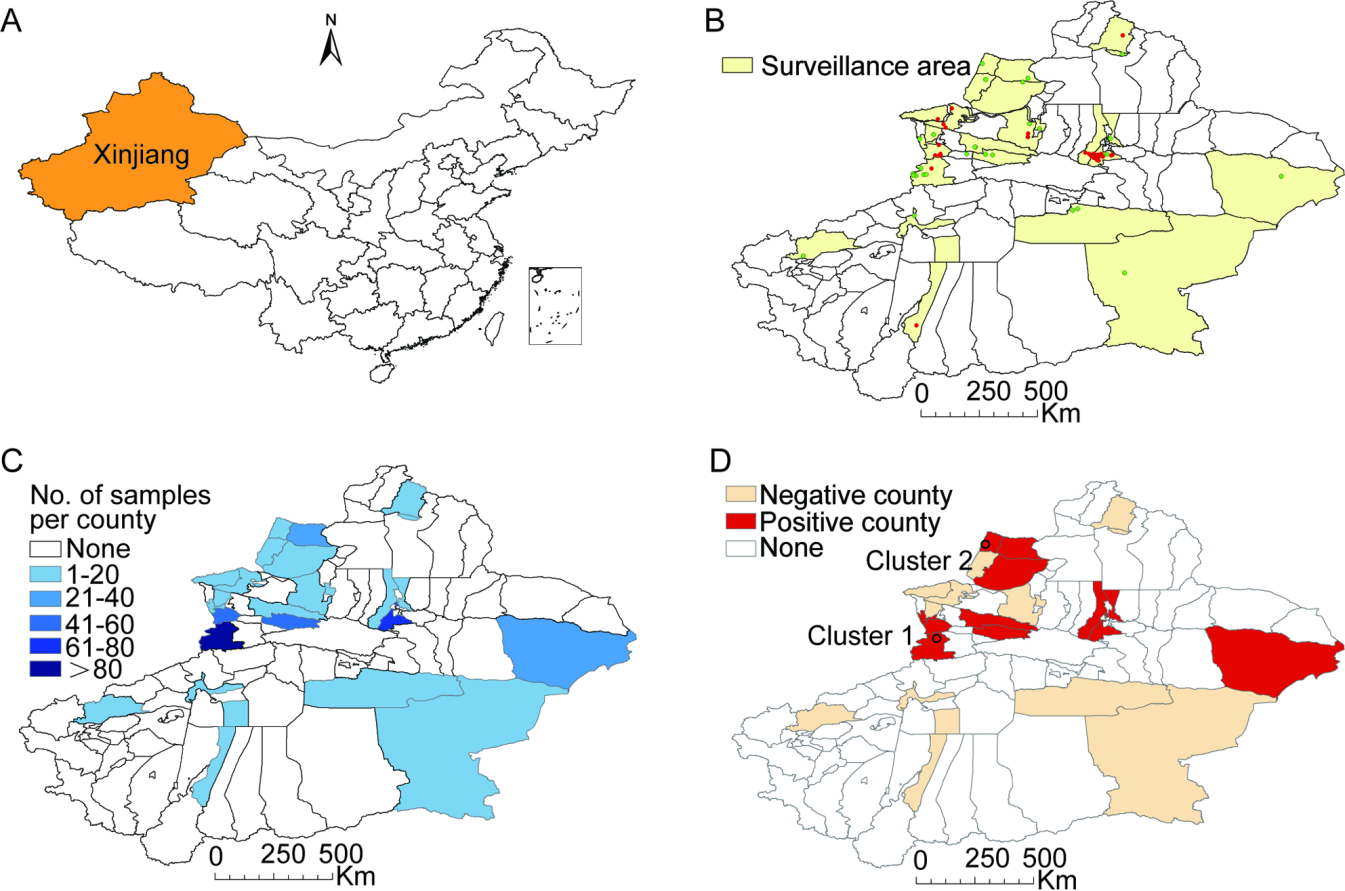
Spatial analysis of TB in wild boars in Xinjiang, China. **A** The location of Xinjiang in China. **B** Surveillance area for TB in wild boars. Red dots indicate the sites where feral wild boars were collected, and green dots indicate the sites where farmed wild boars were collected. **C** Distribution of serum samples analyzed per county. **D** Distribution of TB positive samples. Red circles indicate the two identified clusters

Xinjiang (34°25′N–48°10′N, 73°40′E–96°18′E) is the largest province in China, comprising 106 county-level administrative regions with a total land area of 1,664,900 km^2^ (Fig. 1, panel A). Wild boars were hunted and sampled with the consent of the Xinjiang Forestry and Grassland Bureau and samples were processed according to the Law of People’s Republic of China on the Protection of Wildlife. We collected 512 serum samples from wild boars in 25 county-level administrative regions during 2017–2020, including 391 from farmed wild boars and 121 from feral wild boars (Fig. 1, panel B). Notably, no visible nodules were observed in the lungs and hilar lymph nodes of feral wild boars, and no nucleic acid was detected as positive for MTBC according to the method described by Huard et al. [15]. All samples were transported on dry ice and stored at -80 °C.

To determine the presence of antibodies against *M. bovis*, serum samples were tested for anti-bovine tuberculin purified protein derivative (bPPD, derived from *Mycobacterium bovis*) immunoglobulin antibodies using ELISA with bPPD as antigen and protein G horseradish peroxidase as a conjugate. ELISA plates were coated with 100 µL of bPPD (10 µg/mL) for 18 h at room temperature, and were washed with PBS containing 0.05% Tween 20 (PBST), and incubated for 1 h at 37 °C with 200 µL/well of 1.0% bovine serum albumin (Sangon, Shanghai, China) in PBST to block potential free binding sites. Serum samples (100 µL/well) were added at a dilution of 1:200 in PBS and incubated for 1 h at 37 °C. Protein G-horseradish peroxidase conjugate (Invitrogen) was added (100 µL/well) at a dilution of 2.5 µg/mL in PBST and incubated at 37 °C for 1 h. A total of 100 µL of SigmaFast OPD substrate (Sigma-Aldrich, USA) was added to each well and incubated at room temperature in the dark. The reaction was stopped after 20 min by the addition of 50 µL/well of 3 N H_2_SO_4_. The optical density (OD) was measured by using a spectrophotometer at 450 nm. Each plate included duplicates of blanks, as well as positive and negative controls, along with the samples. The cutoff value was calculated as mean sample OD450nm ≥ 2×the mean negative control OD450nm.

Chi-square test was used for analyzing the relationships between different feeding modes. The results of risk estimation are presented as odds ratio (OR) with corresponding 95% confidence interval (CI). All the statistical analyses were performed using SPSS v26. A two-tailed p value < 0.05 was used to indicate statistical significance.

We obtained serologic samples for wild boars in 25 out of 106 counties. We constructed a regional distribution map with differences in coloring to indicate the numbers of samples collected in different areas and performed spatial interpolation using ArcGIS v10.2 (Fig. 1, panel C). For spatial distribution, we employed cluster analysis with purely spatial scan statistics using the Bernoulli model and setting the maximum cluster size at 50% of the population at risk with SatScan v9.6 software [16].

## Results

We detected antibodies against bPPD in 51 out of 512 wild boars (10.0%, 95% confidence interval (CI) 7.4%–12.6%). Antibody-positive samples originated from 12 out of the 25 counties tested, ranging from 3.8% (95%CI 0–7.9%) in Urumqi county to 60.0% (95% CI 29.6%–90.4%) in Qapqal county, with no seropositive samples detected in 13 counties (Table 1).

**Table 1:** The detection results of tuberculosis in wild boars

County*	No. tested	No. seropositive (%, 95% CI)
Aksu	5	0
Altay	3	0
Artux	4	0
Beitun	8	0
Bole	8	0
Qapqal	46	2 (4.3, 1–15)
Changji	6	1 (16.7, 0–64)
Emin	40	6 (15.0,6–30)
Huocheng	10	0
Kokdala	5	1 (20.0, 1–72)
Kuitun	6	0
Luopu	2	0
Midong	15	1 (6.7, 0–32)
Nilka	9	2 (22.2, 3–60)
Ruoqiang	7	0
Tacheng	10	6 (60.0, 29.6–90.4)
Tuoli	20	1 (5.0, 0–25)
Yuli	12	0
Wenquan	1	0
Urumqi	80	3 (3.8, 0–7.9)
Wusu	14	0
Xinyuan	56	7 (12.5, 3.8–21.1)
Yizhou	21	3 (14.3, 3–36)
Yumin	20	0
Zhaosu	104	18 (17.3, 10.0–24.6)
Total	512	51 (10.0, 7.4–12.6)

* including counties, county-level cities or districts in current administrative divisions of China

To evaluate differences in feeding modes, we compared feral and farmed wild boars. We detected four out of 121 (3.3%, 95% CI 0.1%–6.5%), and 47 out of 391 (12.0%, 95% CI 8.8%–15.2%) positivity in feral and farmed wild boars, respectively (Table 2). The seroprevalence in farmed wild boars was statistically higher than that in feral ones (χ2 = 7.825，*p* = 0.005). The results indicated that farmed wild boars had a relatively higher risk of TB than feral wild boars (odds ratio (OR) = 4.00, 95% CI 1.41–11.33) (Table 2).

**Table 2:** Prevalence of tuberculosis in different feeding modes

Animal	No. tested	No. seropositive (%, 95% CI)	OR (95% CI)	*p*-value
Feral wild boar	121	4 (3.3, 0.1–6.5)	Reference	0.005
Farmed wild boar	391	47 (12.0, 8.8–15.2)	4.00 (1.41, 11.33)	

Analysis of the spatial distribution revealed two clusters of TB in wild boars. Cluster 1 represented one county (Qapqal county) with 18/104 positive wild boars (relative risk = 2.14, *p* = 0.189), and cluster 2 represented one county (Tacheng county) with 6/10 positive wild boars (relative risk = 6.69, *p* = 0.002). These two clustering sites are located adjacent to the Sino-Kazakh border (Fig. 1, panel D).

## Discussion

To our knowledge, this study represents the first serological investigation of TB prevalence in both feral and farmed wild boar populations in China, employing a bPPD-ELISA method that has been specifically validated for wild boars. While serological performance for TB can be variable in other mammals, this assay demonstrates good specificity and moderate sensitivity in this species [17, 18], making it a reliable tool for large-scale serosurveillance. Our findings provide crucial baseline data for understanding the epidemiology of TB at the wildlife-livestock interface in China. The application of this validated serological approach to wild boars, recognized as important sentinel species for wildlife TB monitoring, offers valuable insights for developing national surveillance strategies.

The bPPD antigen used in this study is derived from *Mycobacterium bovis* and exhibits cross-reactivity with antibodies against other members of the MTBC due to antigenic similarities [19]. While this characteristic makes the serosurvey a valuable tool for detecting broad MTBC exposure, it limits the ability to distinguish the specific causative species. It is possible that infections caused by less common or regionally prevalent MTBC members, such as M. orygis (which is epidemiologically significant in South Asia) [20], could demonstrate variable seroreactivity. Therefore, future studies should incorporate molecular characterization of isolates to identify the specific MTBC species circulating in wild boar populations in Xinjiang and to clarify the serological cross-reactivity patterns.

Wild boars are of significant ecological and epidemiological concern as potential reservoirs for the MTBC, especially considering their unique biological characteristics and population dynamics [12, 21]. Originally introduced as game animals, wild boars have demonstrated remarkable adaptability, with population growth driven by multiple factors including their omnivorous feeding habits, high reproductive capacity, absence of natural predators, and stringent wildlife protection laws [11]. This population expansion, coupled with their increasing proximity to human settlements and agricultural areas, creates multiple pathways for potential disease transmission. Of particular concern is the genetic introgression between wild boars and domestic pigs through interbreeding, which may facilitate pathogen spillover across species barriers [22].

Our findings have uncovered significant epidemiological patterns that demand urgent attention. The higher detection rate of TB in farmed wild boars compared to feral wild boars is likely attributable to elevated population densities in farmed conditions and the consequent increased potential for pathogen transmission in managed environments [23, 24]. Moreover, the identification of two distinct TB clusters near the Sino-Kazakh border highlights the critical importance for international collaboration in disease surveillance and control. Although the clusters’ proximity to the border suggests possible transboundary transmission, the existence of undetected domestic links—such as through intra-regional animal movements or trading networks within Xinjiang—cannot be excluded. Future studies that integrate molecular epidemiology with detailed farm trade tracing are needed to elucidate the transmission dynamics among these and any future clusters. This approach is especially pertinent given the extensive cross-border movements of wildlife and the ecosystems shared between China and Central Asian countries. These findings underscore the necessity of strengthened cross-border cooperation to effectively mitigate the threats posed by zoonotic diseases in this region.

The detection of MTBC in Chinese wild boar populations is consistent with previous reports from Europe and South Korea [25, 26], where wild boars have been identified as significant reservoirs for TB. It is noteworthy that no macroscopic tuberculous lesions were observed in the lungs or hilar lymph nodes in our study. This finding aligns with reports such as that by Martín-Hernando et al. [27], which indicate that visible lesions in infected wild boars are frequently localized in the head and mandibular lymph nodes, while lung-associated pathology often only becomes apparent upon histopathological examination. The absence of gross lesions in our samples may thus be attributable to this distinct lesion distribution rather than a true absence of infection. This highlights the potential for interspecies transmission, particularly given the frequent interactions between wild boars and domestic animals in agricultural settings [28]. Our results underscore the urgent need to develop and implement quarantine protocols specifically designed for wild boar populations, as current practices predominantly focus on cattle and humans. Such measures should be complemented by integrated control strategies including: enhanced surveillance of wild boar populations in high-risk areas, development of targeted intervention programs for farmed wild boars, implementation of environmental decontamination protocols in endemic areas, establishment of regional collaboration frameworks for disease monitoring and development of public awareness campaigns about wildlife disease risks. These integrated control strategies are essential to mitigate the threat posed by MTBC in wild boar populations and to protect both human and animal health.

The epidemiological implications of our findings extend beyond wildlife management, underscoring the necessity for a One Health approach to TB control. The potential for environmental contamination through shedding of MTBC by infected wild boars poses significant challenges. The pathogen’s ability to persist in soil and water for extended periods creates persistent reservoirs that can facilitate subsequent transmission to domestic animals and humans [29]. This highlights the importance of integrating veterinary, public health, and environmental strategies to effectively mitigate the risks associated with TB in wild boar populations and their surrounding ecosystems.

In conclusion, this study provides serologic evidence supporting the role of wild boars as potential reservoirs of MTBC in China, thereby highlighting the critical importance of integrating wildlife health monitoring into national TB control programs. Our findings emphasize the need for regular screening of wild boar populations, development of species-specific diagnostic tools, implementation of targeted control measures, and strengthening of international collaboration for disease surveillance. Future research should focus on molecular characterization of MTBC strains in wild boars, quantitative risk assessment of transmission pathways, and evaluation of intervention strategies. These efforts are essential for developing effective management strategies to mitigate TB transmission at the wildlife-livestock-human interface, ultimately contributing to the global goal of TB eradication.

## Data Availability

The datasets and materials used and/or analysed during the current study are available from the corresponding author upon reasonable request.
